# Motivational interviewing for genetic counseling: A unified framework for persuasive and equipoise conversations

**DOI:** 10.1002/jgc4.1609

**Published:** 2022-07-30

**Authors:** Ken Resnicow, Emerson Delacroix, Gabriela Chen, Sarah Austin, Elena Stoffel, Erika N. Hanson, Lynette Hammond Gerido, Kimberly A. Kaphingst, Beverly M. Yashar, Monica Marvin, Jennifer J. Griggs, Deborah Cragun

**Affiliations:** ^1^ University of Michigan School of Public Health Ann Arbor Michigan USA; ^2^ Rogel Cancer Center University of Michigan Ann Arbor Michigan USA; ^3^ Department of Internal Medicine University of Michigan School of Medicine Ann Arbor Michigan USA; ^4^ Genetic Counseling Graduate Training Program, Department of Human Genetics University of Michigan Ann Arbor Michigan USA; ^5^ Department of Communication University of Utah Salt Lake City Utah USA; ^6^ Huntsman Cancer Institute University of Utah Salt Lake City Utah USA; ^7^ University of South Florida, College of Public Health Tampa Florida USA

**Keywords:** counseling techniques, decision‐making, education, professional development

## Abstract

Genetic counselors (GCs) have traditionally been trained to adopt a position of equipoise or clinical neutrality. They provide information, answer questions, address barriers, and engage in shared decision‐making, but generally, they do not prescribe a genetic test. Historically, GCs have generally been trained not to persuade the ambivalent or resistant patient. More recently, however, there has been discussion regarding when a greater degree of persuasion or directionality may be appropriate within genetic counseling (GC) and what role MI may play in this process. The role for “persuasive GC” is based on the premise that some genetic tests provide actionable information that would clearly benefit patients and families by impacting treatment or surveillance. For other tests, the benefits are less clear as they do not directly impact patient care or the benefits may be more subjective in nature, driven by patient values or psychological needs. For the former, we propose that GCs may adopt a more persuasive clinical approach while for the latter, a more traditional equipoise stance may be more appropriate. We suggest that motivational interviewing (MI) could serve as a unifying counseling model that allows GCs to handle both persuasive and equipoise encounters. For clearly beneficial tests, while directional, the MI encounter can still be non‐directive, autonomy‐supportive, and patient‐centered. MI can also be adapted for equipoise situations, for example, placing less emphasis on eliciting and strengthening change talk as that is more a behavior change strategy than a shared decision‐making strategy. The core principles and strategies of MI, such as autonomy support, evocation, open questions, reflective listening, and affirmation would apply to both persuasive and equipoise encounters. Key issues that merit discussion include how best to train GCs both during their initial and post‐graduate education.

## 1 INTRODUCTION

Genetic counseling (GC) is centered around the principle that testing decisions should be patient driven. A goal of genetic counseling is to arrive at autonomous and fully informed choices about whether to undergo genetic testing (GT) and what action to take based on test results. To this end, GCs have historically been trained to adopt a position of equipoise or clinical neutrality. That is, they provide information, answer questions, address barriers, and engage in shared decision‐making but generally do not provide strong recommendations or directive advice. The reluctance to prescribe a genetic test is driven by both ethical concerns (to avoid coercion) and scope of practice (GCs cannot give medical advice). Also, GCs largely assume that patients have the competence to make informed testing decisions. Language such as “I strongly recommend you get this test, “You should definitely get this test,” and “It’s important that you get this test,” is generally discouraged as it is seen as overtly directive and a threat to patient autonomy. While GCs may communicate that a patient is eligible for testing based on clinical guidelines and they support patients who express interest in testing, they rarely try to convince an ambivalent or resistant patient to get tested (Redlinger‐Grosse, 2020).

More recently, there has been increased discussion regarding a potential role for incorporating more persuasion or directionality within GC, around both proband testing as well as family communication and cascade testing (Ash, 2017; de Geus et al., 2016; Kinney et al., 2018; Kruger et al., 2019; Winchester et al., 2022). The rationale for “persuasive GC” is based on the premise that some genetic tests provide actionable information that would clearly benefit patients and families. This is analogous to how primary care medical providers (PCPs) approach issues such as smoking cessation and diabetes management (Ash, 2017). Because there is a clear medical benefit for almost all patients to quit smoking or lower elevated blood glucose levels, PCPs are encouraged to proactively broach these issues with their patients and help motivate them to change (Puschel et al., 2008; Sturgiss et al., 2022). While there is general agreement that such conversations should remain patient‐centered and be devoid of coercion, guilt, shame, or external pressure, these encounters are nonetheless directional and goal driven. The provider does not offer continuing to smoke or maintaining elevated A1c levels as equally preferred options. On the other hand, there are many medical decisions where persuasion is less appropriate and a position of equipoise is called for, either because there is no clear‐cut benefit to the patient (e.g., prostate cancer screening for individuals at average risk), there are multiple options with similar benefit (e.g., stool DNA testing versus colonoscopy for colon cancer screening among individuals at average risk), or the decision is related to patient values and preferences rather than medical benefit (e.g., having breast reconstruction after removing a malignant breast tumor) (Jamal et al., 2020).

The same distinction between *persuasive* and *equipoise* encounters found in primary care medicine can be applied to GC (Ash, 2017). Some genetic tests are clearly beneficial for medical management of patients and their relatives, and for these instances, a more directional or persuasive approach to GC may be warranted. This category would include tests for hereditary cancer syndromes (e.g., hereditary breast‐ovarian cancer [HBOC] and Lynch syndromes), cardiomyopathies, and familial hypercholesterolemia (Miller et al., 2021). The actionable benefits in these cases can include precision treatment and surveillance regimens, which can impact morbidity and/or mortality for the proband and their relatives (Owens et al., 2019; Syngal et al., 2015).

Alternatively, there are GTs for which the benefit is less clear as testing does not impact screening, surveillance, or treatment or because there is higher variability in expressivity or penetrance (e.g., Huntington's disease or late‐onset Alzheimer’s disease). These genetic tests would fall under the “equipoise” class.

There is also perhaps a third class, a gray area, that lies in between the persuasive and equipoise domains. Some of the “equipoise” tests mentioned above may yield psychological benefits for some patients and their family members. For example, by knowing genetic carrier status, some patients may be better prepared cognitively and emotionally to handle the onset or progression of symptoms or they can make informed decisions around family planning. Additionally, knowing one’s genetic status may allow some patients to enroll in clinical trials or other research that could help future generations, which for some serves as a psychological benefit. Applying this broader definition of benefit could shift some preference‐sensitive and “gray” tests to the persuasive side of the ledger, while a more conservative approach would be to treat such cases with equipoise.

In addition to psychologic gray areas, there are gray areas of medical benefit. Consider a pregnancy with multiple congenital anomalies where undergoing prenatal testing can impact care plans (e.g., delivering in a hospital with a specialty care nursery, options for in‐utero interventions). Although there may be medical benefits to identifying a prenatal diagnosis in this situation, discussion of testing needs to be balanced with risk for miscarriage with invasive testing (CVS/Amnio), psychological harm, and patient values. The same testing offered as a screening test in an otherwise average‐risk healthy pregnancy would likely fall into the equipoise category.

So too in the hereditary cancer context, there are gray areas as to what is considered actionable. Screening for some cancer predisposition genes may have conflicting or limited evidence regarding increased screening (e.g., *MUTYH, NBN, RAD50),* or have the potential to be actionable only in the context of specific family history (e.g., pancreatic cancer screening for *ATM* pathogenic variant carriers). For the highly penetrant inherited cancer syndromes (HBOC/Lynch), genetic testing could be considered preference‐sensitive when patients do not qualify for genetic testing based on established guidelines (e.g., National Comprehensive Cancer Network, NCCN) even though identification of these syndromes would be actionable. Another gray area would be in men with a family history of early‐onset breast cancer who meet clinical criteria for testing, but the actionable impact on surveillance (standard PSA levels with added clinical breast exams) is only marginally different than the general population. A woman in the same scenario may be recommended to adopt substantial changes to their screening, surveillance, and treatment regimens if a pathogenic variant is found. Thus, the boundary between persuasive and equipoise encounters can be ambiguous, and perhaps the equipoise versus persuasive distinction may better be represented along a continuum rather than discrete categories.

Ultimately, whether or not a genetic test result might be considered beneficial and actionable may depend on subjective judgments on the part of the patient, for example, values or preferences such as pregnancy termination following prenatal testing. As a guiding principle, we propose, for these subjective benefit scenarios, these gray areas, that the counselor should generally remain in equipoise rather than persuasive mode.

Whereas what constitutes benefit may be open to debate among genetics professionals and may depend on patient‐specific factors (preference‐sensitive), for purposes of this paper, we classify as persuasive those tests that have a reasonable chance of finding a pathogenic variant that would substantially impact medical treatment or surveillance for the proband or their family members and there is substantive evidence that those treatments or surveillance regimens can positively benefit their health. No doubt, which tests fall under each category will likely shift as new genetic markers, treatments, and diagnostic tools become available. Nonetheless, based on the current state of the science, we provide examples of both persuasive and equipoise scenarios in Table 1. While which column specific genetic tests fall under is important, our goal in this paper is not to definitively classify all tests into their level of benefit. Instead, we intend to illustrate that different counseling approaches and skills are indicated for different scenarios or types of testing and propose that persuasive encounters can be conducted differently than preference‐sensitive encounters. Note, we address only behaviors directly related to genetic testing and have not included behaviors such as quitting smoking, age‐related cancer screening, and managing a chronic disease that may have clear‐cut benefits and would fall under the persuasive column but are not the primary focus of GC.

**TABLE 1:** Examples of persuasive and equipoise genetic counseling scenarios

Persuasive GC: Clear cut medical benefit	Equipoise GC: Preference‐sensitive subjective benefit
Genetic testing for highly penetrant hereditary cancer syndromes (hereditary breast ovarian cancer, Lynch syndrome, familial adenomatous polyposis [FAP], Peutz‐Jegher syndrome, Li Fraumeni syndrome, etc)	Prenatal genetic screening or testing for chromosomal aneuploidy (e.g., NIPT, Amniocentesis, CVS)
Genetic testing for familial hypercholesterolemia	Preconception carrier screening
Universal newborn screening	Prenatal testing for family planning decisions including gamete donation, preimplantation genetic testing, adoption, continuing pregnancy vs pregnancy termination
Genetic testing for inborn errors of metabolism in symptomatic individuals	Genetic testing for Huntington Disease and Parkinson’s Disease
Informing family members regarding known hereditary conditions	Population screening for healthy individuals
Genetic testing for inherited cardiomyopathies, aortopathies, and arrhythmias	Receiving secondary findings on whole exome sequencing
Genetic testing for infants presenting with multiple congenital anomalies	Genetic testing using multi‐gene cancer panels with preliminary evidence/limited evidence genes

a classification as beneficial assumes the patient meets clinical testing guidelines (e.g., NCCN (Daly et al., 2021), ACMG ((American College of Medical Genetics and Genomics, 2022); Miller et al., 2021) based on personal or family history.

### 1.1 Counseling for persuasive and equipoise encounters

This background brings us to our central thesis, which is, persuasive and equipoise GC encounters require different counseling styles, skills, and strategies. Whereas GCs are generally well‐trained to handle equipoise encounters and to evoke shared decisions, they have generally not been trained to use more persuasive techniques. Yet, the GC discipline may benefit from an adaptive framework where counselors can adjust their style depending on the type of patient they face, and as Ash notes, perhaps even within the same patient encounter depending on what decision is being discussed (Ash, 2017). Current practice guidelines already call for “adapting genetic counseling skills for varied service delivery models.” See Competence 12 of the American Board (Accreditation Council for Genetic Counseling, 2019; American Board of Genetic Counselors, 2022). We propose that motivational interviewing (MI) can serve as a unifying clinical model that can be used for both types of GC scenarios. As we will describe, MI can be used for directional, persuasive encounters as well as equipoise, shared decision‐making, while in both instances, remaining autonomy‐supportive and patient‐centered. In other words, MI aligns with the principles and practice of genetic counseling.

### 1.2 Motivational interviewing (MI)

We have chosen MI as the clinical model for several reasons. First, having a defined model for conducting GC encounters can provide guidance with regard to training GCs, quality control, and identifying what psychosocial mediators to target. Second, MI has shown to be effective in promoting behavior change over hundreds of trials, across multiple health behaviors, among a wide array of clinicians and clinical contexts (Hall et al., 2016; Hettema & Hendricks, 2010; Lindson‐Hawley et al., 2015; Lundahl et al., 2010; Lundahl et al., 2013; Palacio et al., 2016; Zomahoun et al., 2017). Second, both the spirit and practice of MI allow it to be adapted for use in both persuasive and equipoise encounters. Like GC, MI is deeply rooted in principles of autonomy support, patient‐centeredness, and non‐directiveness, regardless if the encounter is persuasive or preference‐sensitive in nature. MI can be *directional* without being unduly *directive*.

Finally, MI aligns well with ACGC practice guidelines (Accreditation Council for Genetic Counseling, 2019) and the National Society of Genetic Counselors Task Force report (Resta et al., 2006) that specifically mention principles and practices akin to those found in MI. In fact, many GCs are already using some MI techniques and may operate from a similar conceptual framework.

While most MI‐based encounters are non‐directive, under some conditions (e.g., direct request from a patient for guidance) the MI counselor can offer clear advice. Thus, the GC’s recommendation whether or not to get a particular test, can be autonomy supportive. An example is shown in supplement 1. Importantly, as we will describe later, different MI skills and strategies may be applicable across the different types of GC encounters (Ash, 2017; Baldry et al., 2021; Kruger et al., 2019; Winchester et al., 2022). We now describe MI and then discuss how it can be implemented within the context of both persuasive and equipoise GC encounters.

### 1.3 Defining Motivational Interviewing (MI)

The widely accepted, short definition of MI is “a collaborative, person‐centered form of guiding to elicit and strengthen motivation for change (Miller & Rollnick, 2013).” Perhaps, the most consequential word in this definition is “change” (Miller & Rollnick, 2013). MI is a behavior change technique not just a method for establishing rapport and expressing empathy. It is at its core persuasive. While goal‐oriented and *directional,* it is rarely *directive*. Directive can be distinguished from directional in several ways. First, directive refers to the style or tone of communication employed by the practitioner, whereas directional refers to the nature or context of the encounter itself. A directional encounter has a clear goal. In the case of smoking cessation, for example, the implicit directional goal is to reduce or quit smoking (rather than increase it). For persuasive genetic testing encounters, the assumed direction is toward getting tested. This can be distinguished from equipoise encounters where there is no assumed direction. Even when there is an assumed direction to the conversation and the provider uses persuasive techniques, the tone of the encounter remains autonomy supportive. A directive approach, which generally entails the provider driving the decision and using more controlling (non‐autonomy supportive) language, is rarely recommended. Importantly, as discussed later, MI, with some adaptation, can be used for both persuasive and equipoise encounters. First, we describe the essential principles and techniques of MI, and then discuss how it is used in the context of persuasive encounters.

Motivational interviewing is a counseling style initially used to treat alcohol misuse and other addictions (Heather et al., 1996; Kadden, 1996; Miller, 1983; Miller & Rose, 2009; Rollnick et al., 1992). The first paper on MI was published by William Miller in the mid‐1980s (Miller, 1983), and since then it has been applied across a wide range of health conditions beyond substance misuse including behavioral health and the prevention and management of chronic diseases. Change, while more commonly thought of in terms of reducing a risk behavior like substance misuse or adding a protective behavior such as physical activity, could also apply to undergoing genetic testing. There has been some conceptual work discussing the role of MI for GC (Ash, 2017) and some qualitative and pilot studies in the GC realm, largely focused on familial hypercholesterolemia and cancer (de Geus et al., 2016; Kinney et al., 2014; Kruger et al., 2019; Razo‐Mejia et al., 2014; Resta et al., 2006; Schwartz et al., 2014; Winchester et al., 2022).

MI comprises both relational and technical components. The relational (some view as the philosophical) elements include autonomy support, empathy, affirmation, collaboration, and respect for the patient. The technical component includes specific skills and strategies many GCs may already employ such as open questions, reflective listening, shared agenda setting, evocation, summarizing, and eliciting change talk. An effective MI practitioner is able to strategically navigate between “comforting the afflicted” and “afflicting the comfortable,” to balance the expression of empathy with the need to build sufficient drive or discrepancy to stimulate change.

A core goal of MI is to assist individuals to work through their ambivalence or resistance (more recently referred to as discord) about behavior change and to generate their own argument for taking action. The tone of MI is non‐judgmental and affirming. Counselors establish a non‐confrontational and supportive climate in which patients feel comfortable expressing the reasons for and against taking action. Ambivalence is explored prior to moving toward action, that is, the *why* component is explored before proceeding to the *how* component, where goals are set and a plan of action is developed.

In MI much of the psychologic work, that is, problem‐solving and action planning is done by the patient. Guided, and at times evoked, by the counselor, patients generate their own rationale for change. Directive advice is rarely used and suggestions and information are generally conveyed using the Elicit‐Provide‐Elicit approach, discussed below. Unlike cognitive‐behavioral interventions (Miller & Rollnick, 2009), MI counselors generally do not attempt to counter message resistance or confront irrational or maladaptive beliefs. Instead, they may subtly help patients detect possible contradictions in their thoughts and actions leading patients to experience discrepancies between their current actions and their broader values. In the case of genetic testing, this may include connecting testing to feeling more in control of their health, or feeling responsible, respected, and admired by family members with whom they may share their testing results.

### 1.4 Theoretical underpinnings

MI arose from intuitive clinical practice rather than any particular theoretical model. It emerged in part as an alternative to the directive, even confrontational style of substance use counseling commonly used during the 1980s (Miller & Rose, 2009). Many of its principles and techniques are rooted in the patient‐centered approach of Rogers and Carkauff, although MI is more goal‐driven and directional than classic Rogerian therapy (Carkhuff, 1993; Carkhuff et al., 1979; Miller & Rollnick, 2009; Rogers, 1986). Despite MI’s largely atheoretical origins, many MI researchers and practitioners use self‐determination theory (SDT) to understand how and why MI works (Markland et al., 2005; Resnicow & McMaster, 2012; Vansteenkiste & Sheldon, 2006).

Originally proposed by Deci and Ryan, SDT conceptualizes a continuum of human motivation, ranging from amotivated (I cannot even think about it) to fully autonomous (I want to do this because it is meaningful and manageable; Deci et al., 1999; Ryan et al., 1997; Ryan & Deci, 2000). Autonomous motivation, in which the patient finds meaning in the behavior and acts with volition, is seen as a more powerful and lasting pathway than controlled motivation where the patient acts out of external pressure such as from loved ones, family members, or health care providers, or internally voiced shame or guilt (Ng et al., 2012). Thus, SDT distinguishes between different qualities of motivation. In the context of GC, autonomous motivation might entail a patient expressing a desire to get tested based on their own volition and because they believe it will help them stay in control of their health care and help family members manage their risk. That is, they find positive meaning in getting tested.

Self‐determination theory similarly proposes three fundamental human needs that are relevant to GC: autonomy, competence, and relatedness. SDT research has shown that, when these needs are met, people experience higher quality motivation as well as more successful behavior change, greater psychological well‐being, and higher post‐decision satisfaction (Ng et al., 2012; Shumway et al., 2015). From an SDT perspective, genetic counseling that supports these needs and is volitional, aligned with patient values, and consistent with their perceived competence will be more likely to be positively appraised than testing decisions that are driven by more controlled regulation. On the other hand, counseling that does not support these three needs, which would include directive persuasion that is not autonomy supportive, will lead to worse outcomes both in terms of the testing decision itself as well as the appraisal thereafter.

Another principle of SDT that relates to motivational interviewing is that motivation can be conceptualized as how much energy the patient is willing and able to invest in the behavior (change). For GC, this may include how much energy the patient is willing to devote to understanding their need for testing and how much energy they have available to understand their results, communicate them with family, and act on them. Resistance then can be thought of as the energy expressed by the patient against change. Often resistance is driven by a fear on the part of the patient that they do not have enough energy to handle the challenge at hand or that they already feel overwhelmed by the task. In the case of GC, this may relate to being overwhelmed with their recent diagnosis or the fear of having to absorb a lot of new information about genetics and how to interpret their results.

Understanding GC through the lens of SDT requires delineating a distinction between autonomy and independence (Resnicow et al., 2021; Vansteenkiste et al., 2012). Not all GT decisions have to be driven by the patient to be autonomous. Autonomy is central to SDT and GC, but it is not synonymous with independence. Independence refers to how much the patient acted on their own behalf without input from others. In SDT the opposite of independence is dependence. Dependence occurs when a patient relies on the advice and help of their provider to resolve their ambivalence and determine a course of action. A patient who decides to get GT on their own, with little input from a provider, functions independently, while the patient who wants guidance or advice from their GC or physician regarding their decision displays *volitional dependent* functioning. However, both the independent and volitionally dependent patient can still be *autonomous* (Resnicow et al., 2021). A patient may autonomously decide to seek their health care provider’s input or direction about GT, that is, they may decide autonomously to be non‐independent. Thus, in some instances, a GC may be asked by the patient to provide some direction. The goal is for clinicians to adapt their counseling style to meet the needs and preferences of the patient; to titrate their directiveness. However, even in situations where the GC is asked to provide direction or a clear recommendation, it is still important to remain autonomy supportive by conveying to the patient that the final decision is up to them, and the provider will not abandon them if they decline to get tested. Finally, relatedness, the third of the SDT basic needs involves the desire to form meaningful social connections. In GC this can include the rapport between patient and counselor as well as how the proband getting tested or sharing their results with family has a positive impact on their relationships.

The essence of MI lies in its spirit; however, specific techniques and strategies help ensure such spirit is evoked. To this end, counselors using MI rely heavily on techniques such as open questions, reflective listening, summaries, eliciting change talk, and Elicit‐Provide‐Elicit (also called Ask‐Tell‐Ask).

**Open questions** are generally preferred to closed questions as they cast a broader net and do not lead the patient into a “yes/no” response. Samples of common open and closed questions can be found in Appendix S1. Questions such as w*hat*, *if any*, *when*, *if ever*, *how*, and *if at all*, are helpful as they can normalize the responses *never*, *not at all*, and *none*, thereby reducing potential defensiveness on the part of the patient. While open questions are typically preferred, some types of close questions can be evocative and function as quasi‐open questions such as, “Do you want any more information about your genetic test results?” or “Did the conversation with your kids about the test go okay?”

**Affirmations** are counselor statements that acknowledge something positive about the patient. This could include an attribute they possess or any attempts they have made at change, even if not successful. Affirmations often take the form of reflections. They can help communicate that the counselor understands what is important to the patient and that they recognize their strengths and abilities, thereby building rapport.

Affirmations can be used in both equipoise and persuasive encounters, since establishing rapport is essential. In both types of encounters, the genetic counselor could affirm how patients have successfully made difficult decisions in the past and use these successes to build patients' confidence regarding the decision to receive GT, or not.

In a persuasive encounter, affirmations can also be used to reinforce change talk. For example, a patient may indicate that they have been doing some research about genetic tests and are curious to know if they carry a pathogenic variant, although they are still worried about cost. In this case, a GC could affirm, “You took the time and effort to research what genetic tests might be helpful for you, and although you still have some concerns, it seems you are moving in the direction of getting tested.” Affirmations are generally expressed in the second or third person rather than using first‐person statements such as “I am proud” or “I am pleased to hear that.” The latter begins to resemble direct praise, which is generally not used in MI.

**Reflective listening** can be conceptualized as a form of hypothesis testing. The hypothesis can be stated in generic terms as “If I heard you correctly, this is what I think you are saying …” or “Given what you said, you might be feeling xx…” Reflections, particularly by counselors who are new to the technique, often begin with the phrase, “It sounds like….” More experienced counselors often phrase their reflections in a more truncated form, such as “You are having trouble with …”, leaving off the assumed “It sounds like….” The goals of reflecting include demonstrating that the counselor has heard and is trying to understand the patient, affirming the patient’s thoughts and feelings without judgment, and helping the patient continue the process of self‐discovery. Even when reflections are inaccurate, through the act of correcting the counselor, patients may clarify their thoughts and feelings and move the discussion forward. This is sometimes referred to as a productive miss or a “foul tip” (Resnicow et al., 2012).

One of the most important elements of mastering MI is suppressing the instinct to respond to patients with questions or premature advice. Questions can be biased by what the counselor may be interested in hearing about, their worldview, or prior experience, rather than what the patient wants or needs to explore. Premature advice, in turn, can elicit resistance or pseudo‐commitment, where the patient superficially agrees to take action but is not truly committed to do so. This is demonstrated in the GC‐patient encounter dialogue in Appendix S2. Reflecting helps ensure that the direction of the encounter remains patient‐driven. The simplest level of reflection tests whether the counselor understood the content of the patient’s statement. Deeper levels explore the meaning or feeling behind what was said. Effective deeper‐level reflections can be thought of as the next sentence or next paragraph in the story, that is “where the patient is going with it.” Reflections involve several levels of complexity or depth (Carkhuff, 1993). We describe several types of reflections, next. Perhaps, except for change talk, each of the reflection subtypes would be applicable to both equipoise and persuasive encounters. Double‐sided reflections may be particularly applicable to equipoise situations.

### 1.5 Types of reflections

**Content reflections** are used to elicit the basic facts in the patient’s story and can be important when trying to gather background information and build initial rapport. They generally entail paraphrasing what the patient just said but without adding much insight or inference to the patient’s initial statement. To avoid parroting, the counselor slightly changes the patient’s words. These reflections generally require less risk and less inference than the other types. In the context of GC, a content reflection could be, “You had breast cancer last year and had a genetic test which showed a pathogenic variant. You haven’t discussed it with any family members yet.” See Appendix S2 for examples.

More complex reflections entail a greater degree of inference from the counselor and manifest as several subtypes including feeling, meaning, rolling with resistance, omission, double‐sided, and amplified negative, each of which is briefly described below.

**Feeling/meaning reflections** often take the form of “You are feeling xx about xx or because of xx.” Meaning reflections may also include a statement about why the person feels a certain way, the symbolic meaning of a behavior or emotion, or how a feeling or action may be related to other important aspects of the person’s life. Often practitioners are reluctant to use emotionally intense words. Glossing over or minimizing patient feelings can communicate counselor discomfort with emotional intensity and shut the patient down. Conversely, acknowledging emotional intensity is a powerful way to quickly build rapport and encourage the patient to fully disclose their thoughts and feelings. An example for GC might be, “you are terrified about finding out whether or not you carry a *BRCA1* gene mutation”, “you dread finding out the result of our test”, “sharing your positive test with family members might make you feel that you are finding something good out of a difficult situation”.

**Rolling with resistance.** Confronting patients can evoke reactance and shut them down. Therefore, MI counselors “roll with resistance” rather than attempt to argue with the patient. Such reflections can be thought of as “comforting the afflicted.” The counselor “pulls up alongside patients,” essentially agreeing with the patient, even if the statement is factually incorrect or unfairly places blame on others. Examples include: “You are worried about the cost of the genetic test” or, “You are so overwhelmed right now with your recent diagnosis that adding a genetic test to the mix feels like it is too much.” Such reflections help capture the patient’s reasons for not changing and allow them to express their resistance without feeling pressured to change or worrying about being judged.

**Amplified negative reflections**. Sometimes rolling with resistance is not sufficient to move the patient forward. When this occurs an amplified negative reflection, that “afflicts the comfortable” may be appropriate. Paradoxically, amplified negative reflections are a way of arguing against change by exaggerating the benefits of or minimizing the harm associated with a risky behavior. It may take the form of “so you see no benefit in changing xx” or “xx is all positive for you.” The counselor, by arguing against change, can exhaust the patient’s negativity. In response, patients will often then reverse their course, and start to argue for change. This type of reflection poses some potential risks, and can occasionally backfire, so it is critical the counselor avoids any tone of sarcasm. This type of reflection is particularly useful when patients appear stuck in a “yes, but” mindset. For GC, it may take the form of “You see absolutely no benefit in finding out your BRCA results” or, “You see no way that you could even broach this topic with family; it seems virtually impossible.”

**Double‐sided reflections** capture patient ambivalence and communicate to the patient that the counselor heard their reasons both for and against change; that the counselor understands the decision is complex, and they are not going to prematurely push them to change. Double‐sided reflections typically take the form of “on the one hand, you would like to change xx, but on the other hand, changing xx would mean giving up xx” or “you are torn about changing xx”. In GC it may be, “On one hand, you are scared how you will handle it if the test comes back positive, but on the other hand you would like to know your genetic status and it might be a relief if you are negative.”

**Reflection of omission.** Sometimes, a counselor can reflect back to patients what they have not said. This can include reflecting on the patient’s silence or reluctance to talk about a particular issue; “You don’t seem like talking about genetic testing today” or “you didn’t have much of a reaction to what I just said”. This can be seen as an extension of rolling with resistance.

A second permutation includes reflecting back to the patient their inferred thoughts on something that has not been directly mentioned but can by its omission be inferred. For example, “You’ve mentioned sharing your FAP results with your sister, but you haven’t mentioned talking with your brother, who appears on your pedigree. You mentioned earlier you aren’t that close. I’m guessing you aren’t all that keen to talk to him about this. For now, we can cross him off the list of people you might talk with”.

**Action reflections** are a key tool in the guiding and choosing phases described later (Resnicow et al., 2012). They incorporate into the reflection possible solutions to the patient’s barriers or a potential course of action. They can be essential in establishing specific action steps for change, in an autonomy supportive rather than prescriptive style. Action reflections may be seen as the bridge between *how* and *why* phases of counseling. They differ from the more common type of reflections such as those that focus on patient feelings, resistance or barriers, or that contain a potential concrete step that the patient has directly or obliquely mentioned. The action reflection looks forward rather than inward or backward. Action reflections contain a possible course(s) that the patient directly mentioned or alluded to. These flow logically from the parameters established by the patient and should not be confused with unsolicited advice. Like any type of reflection, action reflections represent the clinician’s best guess for what the patient has said or more apropos here, where the conversation might be heading.

Action reflections can include multiple choices to support the patient’s autonomy. Resistance to these reflections can be reduced by priming the patient with their own words with a phrase “based on what you said…” or “earlier you mentioned…” or “you’ve considered doing…” The patient is empowered to resolve their ambivalence, enabling them to move toward an action they have considered. For example, “You mentioned some possible options including patient‐initiated testing or requesting your oncologist order one for you.” or “Given what you said about your concerns over cost, it might help for us to talk about what the out‐of‐pocket expense might be for your genetic testing options.” Because the patient directly mentioned or alluded to these possible courses of action, this type of reflection should not be confused with unsolicited advice (something discouraged in MI).

**Change Talk.** A core principle of MI is that individuals are more likely to accept and act upon thoughts and intentions that they voice themselves (Bem, 1972). The more a person argues for a position, the greater their commitment to it often becomes. Therefore, patients are encouraged to express their own (lack of) reasons and plans for change. This process is referred to as eliciting change talk. Expression of change talk, particularly a strong crescendo of commitment, appears to be a good predictor of future change and a key mediator of the MI process. In terms of our “persuasive” versus “equipoise” schema, change talk elicitation applies more to the former and may in fact rarely be used for equipoise encounters.

Confidence‐related change talk may also be nuanced in the context of GC. For example, in changing behaviors such as cigarette use, substance misuse, diet, exercise, et al., a lack of confidence to successfully perform the behavior may be a major factor in patient motivation, and building confidence may be a key intervention goal. For genetic testing, the difficulty may not lie in obtaining the actual test (providing a saliva sample is not in itself particularly difficult) but in understanding the meaning and impact of results and communicating about it with family members.

Eliciting change talk is achieved through several discrete strategies. First, counselors can selectively reflect back change talk, in the hope that such elaboration will move them toward “yes.” An example of a change talk reflection can be found in Supplemental Appendix 2. Another commonly used technique to elicit change talk is the importance/confidence rulers (Resnicow et al., 2015; Rollnick et al., 1997). This strategy typically begins with two questions and is effective for behaviors the patient wants to change: (1) “On a scale from zero to ten, where zero is not at all, and ten is extremely or very, how important is it to you to change [insert target behavior]?” and (2) “On a scale from zero to ten, with zero meaning none at all, and ten being completely, how confident are you that you could (insert target behavior)?” These two questions assess the importance that patients attribute to change and their confidence in being able to change, respectively (Resnicow et al., 2015; Rollnick et al., 1997). Counselors typically follow each of these questions with several probes: “Why not a higher number?” or “Why not a lower number?” to elicit barriers or strengths/values, respectively. For example, the patient has provided several barriers, and lacks confidence for change, they may answer “five.” The counselor would probe first with, “Why did you not choose a lower number, like a three or a four?” followed by, “What might it take to get you to a higher number, like a six or a seven?” These probes elicit change talk and ideas for potential solutions from the patient. Importance, confidence, and readiness can be assessed both at the beginning and end of an encounter.

For use in GC, the important question could be framed as, “On a scale of 0–10, how important is it for you to get the xx test?” or “How important, if at all, is it for you to communicate your xx test results to your family?” The confidence question may require greater adaptation. As noted above, the act of getting a genetic test may in itself not be particularly difficult (swab and mail). However, confidence may be more applicable to understanding test results, explaining them to others, or communicating test results with family members. The questions may be, “On a scale from zero to ten, with ten being the highest, how confident are you that you would understand your genetic test results?” or “On a scale from zero to ten, with ten being the highest, how confident are you that you would be able to explain the results to your relatives?”

A related strategy is to help patients determine how the behavior at hand may align or conflict with other personal values, roles, or goals. This is often accomplished by having the counselor ask the patient to discuss how, if at all, getting genetic testing might impact their ability to achieve roles, goals, or values. In the context of GC, the prompt might be, “How might getting a genetic test impact any of the roles, goals, or values you see in this table?” or “How might any of these values motivate you to get a genetic test?” The patient is typically shown a list of potential roles, goals, and values such as shown in Appendix S3. Potential “linkages” might include that getting tested could aid in feeling in control of their treatment/health, responsible, and respected in the family.

Whereas the roles, goals, and values activity aims to build importance to perform a behavior, sometimes the core issue for the patient is insufficient confidence. In the case of GC this may include confidence to understand and communicate results or confidence to encourage family members to get tested. To build confidence we developed a self‐affirmation (SA) /strengths (SA/S) activity, rooted in self‐affirmation theory (Epton & Harris, 2008) that is a variant of the more traditional values/roles/goals activity noted above.

Here the counselor shows the patient a list of potential strengths, skills, or accomplishments, and says something along the lines of, “Think for a minute about some of the things you are good at, like sports, being a father, art, or meeting challenges at work or something you have achieved, or an obstacle you have overcome.” See Appendix S4. Given the almost limitless universe of strengths, skills, and accomplishments, we suggest adding a statement similar to, “Feel free to suggest something that may not be on the list.” Next, the counselor probes with some variation of:

Looking at the strengths, abilities, or accomplishments you picked…

- How might your success in processing complex information, possibly help you find the confidence to communicate your genetic results with your family?
- How might your ability to research things, listen to others, or stay positive, possibly help you understand your genetic test results?

As a general guideline, we recommend using the values strategy when importance scores on the rulers are lower than confidence scores, while we recommend using the strengths strategy when confidence scores are lower than importance ruler scores. Doing both is possible, but can feel redundant to the patient, as several items appear on both the values and strengths list.

**Summaries** can be used at several junctures in the GC encounter. Toward the end of a session they can be used to summarize what has been said and what, if any, goals or action plans the patient has developed. A GC might include where, when, and how they may obtain their test or how they may communicate their results with family. Summaries can be used mid‐encounter to communicate to the patient that the counselor is tracking the story correctly. Summaries can also be used at key inflection points, such as after change talk has been explored the counselor may selectively recount the benefits for getting a test, while acknowledging barriers or sustain talk as needed, and then evoking from the patient their intentions with something akin to “…having said that, I am curious where that leaves you regarding getting a genetic test”. The latter type of summary would be applicable to persuasive GC more so than equipoise GC.

### 1.6 Elicit provide elicit

Information exchange is a major component of many GC encounters. In MI, information is often presented using the ELICIT‐PROVIDE‐ELICIT framework (Resnicow & McMaster, 2012). This begins by eliciting from patients what they know, what they want to know, and how they want to receive information. After information is provided, the counselor elicits (this is the second “elicit”) what the patient thinks about the information and what, if any, additional information they desire. For GC, it may play out as shown in Appendix S5. The provision of information is generally compressed to a few key facts, and the second elicit allows the patient to receive additional information.

### 1.7 MI for equipoise encounters

So far, we have discussed the application of MI for persuasive GC encounters. Yet MI can, with some adaptation, also be used for preference‐sensitive encounters. In the equipoise context, the spirit or relational aspects of MI remain largely unchanged, that is, autonomy support, empathy, collaboration, and respect for the patient are all still operative. However, as shown in Table 2, some of the technical skills and clinical strategies may be differentially applicable to the persuasive versus preference‐sensitive contexts. For example, under equipoise conditions, the counselor would generally be less focused on evoking and magnifying change talk, as these are more persuasive techniques used to “unstick” the patient and build motivation. Strategies such as the readiness rulers, which are often used to strengthen change talk, can be adapted for equipoise situations by not selectively pulling on change talk responses. This might entail probing both “why not a higher number?” and “why not a lower number?”, rather than focusing on the latter. On the other hand, use of a pros and cons chart and learning assessments, which are often used in shared‐decision making, may be more applicable to equipoise scenarios but less so for persuasive encounters (Matzger et al., 2005). The remaining strategies such as open questions, reflections, summarizing, and Elicit‐Provide‐Elicit would be applicable to both types of encounters. Affirmation can be used in both types of encounters since building rapport, acknowledging effort, and focusing on strengths are important in any MI encounter, even when non‐directional. In the case of persuasive encounters, however, the affirmation may focus more on acknowledging small steps, positive intentions, and other change talk, with the goal of nudging the patient toward testing, that would be less likely to occur in the equipoise scenario.

**TABLE 2:** Motivational interviewing strategies across persuasive and equipoise genetic counseling encounters

MI skill/strategy	Persuasion encounters	Equipoise encounters
Open Questions	X	X
Reflections	X	X
Affirmation	X	X
Summarizing	X	X
Pros and Cons Chart	‐	X
Learning Assessment	‐	X
Elicit Change Talk	X	‐
Readiness Rulers	X	‐
Values and Strengths	X	‐
Elicit Provide Elicit	X	X

Some theorists may posit that when MI is used for preference‐sensitive decisions, it is not truly MI, because there is no clear goal or direction for the encounter. Evocation of change talk becomes largely moot. However, an alternative conceptualization that allows for MI to encompass preference‐sensitive encounters is that, under these conditions, the goal is to achieve a high‐quality decision, which has been defined as the patient feeling they had sufficient time, information, input, and autonomy (Martinez et al., 2016; Resnicow et al., 2014) to make their decision and high satisfaction with their choice.

### 1.8 Training genetic counselors in motivational interviewing

When looking at the Accreditation Council for Genetic Counseling (ACGC) Practice‐Based Competencies for Genetic Counselors (Accreditation Council for Genetic Counseling, 2019), there are four main skill domains: (1) Genetic Expertise and Analysis, (2) Interpersonal, Psychosocial and Counseling Skills, (3) Education, (4) Professional Development and Practice (Accreditation Council for Genetic Counseling, 2019). While MI could likely be incorporated into all 4 domains, there is a clear role for training in MI in Domain 2. Specifically, within Domain 2, MI directly aligns with competency 9; “Employ active listening and interviewing skills to identify, assess, and empathically respond to stated and emerging concerns”; competency 10b, “Utilize a range of basic counseling skills, such as open‐ended questions, reflection, and normalization.”; competency 11.d, “Describe the continuum of non‐directiveness to directiveness, and effectively utilize an appropriate degree of guidance for specific genetic counseling encounters”, and competency 11.b, “Actively facilitate client decision‐making that is consistent with the client’s values.” (Accreditation Council for Genetic Counseling, 2019). Given the natural fit of MI within GC, training in MI may be seen as helping to meet established GC competencies, and therefore less of a major alteration in practice. There are at least a handful of GC programs that already include MI in their training but adding MI to the ACGC competencies could be helpful in encouraging the spread of this evidence‐based practice.

Given the potential fit of MI in GC, a key challenge is how best to train GCs to effectively integrate MI into their clinical repertoire. Many graduate programs outside of GC already include substantial MI training, including those in public health, nutrition, social work, and medicine. This type of training typically entails 2–3 days (the equivalent of 12–16 contact hours) followed by some individual or group supervision using real or simulated patients. Such an approach could fit into modules related to psychosocial counseling skills, particularly courses taught in the second year of training. However, many of the skills described here are basic counseling skills that could be incorporated into first‐year courses and revisited in the second year when addressing more details about other key MI components and concepts and building more advanced MI skills and understanding different theories that guide GC practice. GC programs may already provide some dedicated training to MI but vary in terms of delivery. Some programs may have the option for a semester‐long MI‐elective course, others may have an MI lecture included in their advanced GC theory classes, and others may mention the use of MI in GC without dedicated training in MI skills/methods. At the University of Michigan, we have developed a two‐day intensive MI course tailored to GCs, which has been offered every few years. Student feedback about the workshop has been extremely positive. When not offered, many GC students have attended our broader MI in Public Health course, which includes 15, three‐hour sessions as well as practice with a standardized patient. For GCs already in the field, post‐graduate workshops offering continuing education units (CEUs) may be a feasible option. Workshops can be delivered in‐person or via virtual platforms, led by a human trainer operating in real time or by using self‐guided learning in platforms such as Coursera etc. in which content is pre‐recorded and individuals can practice their skills with online exercises. Hybrid approaches using self‐guided and live interactions represent another option. All of this requires a fundamental acceptance that there is a role for persuasion in genetic counseling, which for some GCs represents a substantial culture shift in how they practice.

## 2 CONCLUSION

Genetic counseling may be facing an inflection point. Historically, the profession has shied away from persuasion, given ethical concerns, practice boundaries, and assumptions about patient independence. However, for some genetic testing decisions, there may be a role for more directional GC. Motivational interviewing can serve as a framework for GCs to deliver both persuasive and more traditional equipoise counseling that remains autonomy supportive and patient‐centered. Many issues remain unresolved, including how best to train GCs during their graduate preparation and those already in the field. The boundary between persuasive GC and medical advice also needs to be defined. What constitutes a persuasive versus preference‐sensitive GC encounter may be a moving target and may depend in part on how heavily psychologic and other more subjective benefits are weighted. Nonetheless, the potential role for the use of MI in GC is promising and additional efforts to train GCs to integrate these skills and examine resulting provider and patient outcomes appear warranted.

## AUTHOR CONTRIBUTIONS

**Ken Resnicow:** Conceptualization; writing – original draft; writing – review and editing. **Emerson Delacroix:** Writing – original draft; writing – review and editing. **Gabriela Chen:** Writing – original draft; writing – review and editing. **Sarah Austin:** Writing – original draft; writing – review and editing. **Elena Stoffel:** Writing – review and editing. **Erika Hanson:** Writing – review and editing. **Lynette Hammond Gerido:** Writing – review and editing. **Kimberly A. Kaphingst:** Writing – review and editing. **Beverly M Yashar:** Writing – review and editing. **Monica Marvin:** Writing – review and editing. **Jennifer J Griggs:** Writing – review and editing. **Deborah Cragun:** Conceptualization; writing – review and editing. All the authors gave final approval of this version to be published and agree to be accountable for all aspects of the work in ensuring that questions related to the accuracy or integrity of any part of the work are appropriately investigated and resolved.

## COMPLIANCE WITH ETHICAL STANDARDS

### Conflict of interest

Ken Resnicow, Emerson Delacroix, Gabriela Chen, Sarah Austin, Elena Stoffel, Erika N Hanson, Lynette Hammond Gerido, Kimberly A. Kaphingst, Beverly M Yashar, Monica Marvin, Jennifer J Griggs, and Deborah Cragun declare that they have no conflict of interest.

### Human studies and informed consent

No human studies were performed by the authors for this Professional Issues paper.

### Animal studies

No non‐human animal studies were performed by the authors for this Professional Issues paper.

### Data sharing and data accessibility

No new data were generated for this Professional Issues paper.

## Supporting information


Appendix S1
