# Supplementary material for: Motivational interviewing for genetic counseling: A unified framework for persuasive and equipoise conversations
Source: J Genet Couns. 2022 Jul 30;31(5):1020–31. doi: 10.1002/jgc4.1609 (PMC9796431; doi:10.1002/jgc4.1609)
Supplement: Supplementary file 1 — Appendix S1 [file JGC4-31-1020-s001.docx]

**Supplemental Appendix 1:** Examples of open and closed questions

| **OPEN** | **CLOSED** |
| --- | --- |
| To what extent…. | Did you..? |
| How often.. | Will you..? |
| Why…. | Can you..? |
| Tell me about….. | Is it…? |
| Help me understand….. | Have you…? |
| What, if any,….. | Didn’t you…? |
| When, if ever, | Could you…? |
| How, if at all, |  |
| What else or, in what ways….. |  |

**Supplemental Appendix 2:** Examples of Reflections for Genetic Counseling

| **GENETIC COUNSELING REFLECTION** | **TYPE OF REFLECTION** |
| --- | --- |
| “You are here today because your oncologist suggested you speak with a GC about genetic testing.”  “You’ve recently been diagnosed with breast cancer and your oncologist referred to our clinic to discuss possibly getting a genetic test” | Content Reflection |
| “You are scared about finding out whether you carry a *BRCA1* mutation” | Feeling Reflection |
| “Your results have valuable information you want to share with your family in hopes of helping others manage their health too.” | Meaning reflection |
| “You are worried about the financial cost of the test as well as how hard it will be to understand the results.”  It’s hard to imagine telling your family they are at risk for having this variant when you haven’t spoken to them in years. | Rolling with Resistance |
| “You see no benefit whatsoever in getting the test for you or your family”  “You see no way that you could even broach this topic with family; it seems virtually impossible.” | Amplified negative reflection |
| “You’re not looking forward to talking with your family about this, but you feel it would be the right thing to do if a gene mutation is found.”  “On one had you want to know once and for all if you carry the gene, but you are not sure how you will handle it if the test show you carry it.” | Double-sided reflection |
| “You haven’t mentioned talking with your brother, who appears on your pedigree. Maybe you don’t seem all that keen to talk to him about this.” | Reflection on omission |
| “You mentioned some possible options including patient-initiated testing or asking your oncologist to order one for you”.  “Given what you said about your concerns over cost, we could talk about what the out-of-pocket expense might be”. | Action Reflection |
| “You see some benefits in getting tested, and your readiness has increased since learning your results can impact your cancer treatment. You are feeling more confident about your ability to handle the test and seem to be moving in that direction.” | Change Talk Reflection |

**Supplemental Appendix 3:** sample list of roles, goals, and values

| good parent  good spouse/partner  responsible  strong  on top of things  competent  athletic  respected at home  energetic  considerate  supportive of others  successful  youthful  genuine | disciplined  attractive  in control  environmentally conscious  respected at work  popular  independent  tolerant  respect for others  justice  community/neighbor  spiritual  not hypocritical  authentic |
| --- | --- |

**Supplemental Appendix 4:** sample list of strengths, skills, and accomplishments

| sports  music  art  cooking  my job  math  science  languages  writing  disciplined  strong  fixing things  trustworthy  parenting  processing complex information | being creative  staying positive  learning new things  staying cool under pressure  being patient  helping others  forgiving  appreciating/being thankful  researching things  listening to others  caring  being spontaneous  beat an illness  other_____________ |
| --- | --- |

**Supplemental Appendix 5:** Standard verses Persuasive MI-based GC encounters

The patient is a 20-year-old cisgender male with a molecularly confirmed paternal family history of familial adenomatous polyposis (FAP), meeting with a GC, in clinic. No previous treatment and no history of colonoscopy, nor other mental or physical health diagnosis. He’s a current undergraduate student, unpartnered, living with peers. The patient’s biological father, age 65, had a colectomy at age 33. MI skills are noted in italics above the GC’s statements. Note that the usual care encounter includes some MI skills.

| Standard GC |  | Persuasive MI-based GC |
| --- | --- | --- |
| GC: “Let me review what I see in your record. Your father had colon cancer at age 33 and tested positive for FAP. It looks like because of that you’ve had a thyroid ultrasound scan a couple years ago that was normal, but you’ve not had a colonoscopy yet. And you haven’t had a genetic test.”  Pt: “Right. I’m just not sure I need that colon test and I don’t want to deal with the genetic test. I was hoping to wait until after college to handle these things.”  *Direction*  GC: “We can talk about why you might want to get the genetic test and colonoscopy sooner rather than later.  Pt: “I know my dad had a bunch of polyps and had to get his colon removed. He said I could also have it.”  *Provide new information*  GC: “Right, FAP causes increased risk for polyps and colon cancer. Based on your father’s diagnosis, we expect you to be at 50% risk to also have FAP.”  Pt: “50/50…that’s a bit better than I thought. I thought it was like 90%”  *Content reflection, provide new information, Closed question*  GC: “Right, there is a chance you don’t have it. Only the test can tell us for sure. Until we have a negative FAP, we recommend regular colonoscopies even at your age. I see you haven’t had a colonoscopy before, right?”  Pt: “No, I haven't. I’m just not sure I need one. I was hoping to wait until after college before I had to deal with this. Plus, the prep sounds terrible.”  *Provide new information*  GC: “I know it might not be the best time with school, but based on your family history and screening guidelines, if you tested positive for FAP we would recommend baseline screening for you starting now. It is important to find and remove any polyps to prevent cancer.”    Pt: “I just don’t know if I am ready to find out if I have it yet.”  *Directive Unsolicited Advice*  GC: “This is a big decision that could impact your health and screening for the rest of your life. It's common for young people in your position to feel this way. But it’s better to know about this sooner rather than later.”  Pt: “Yeah, I can’t imagine having to have a colostomy bag like my dad for the rest of my life.”  *Providing new information*  GC: “Not everyone with FAP needs a colectomy, but it is certainly a possibility especially if there are too many polyps to keep up with. But getting the FAP test is really your call. I don’t like to pressure my patients to get any tests.  Pt: “Maybe I should consider the FAP test more... maybe then I can avoid all these colonoscopies. Maybe I should stop putting it off”  *Content reflection, closed-ended question that works as an open-ended question.*  GC: “So it sounds like you understand the benefits of testing. Do you have other questions about getting tested?”  Pt: “Yeah, how do I get tested?”  *Providing new information*  GC: “If you wanted to move forward with testing, I could mail you a saliva kit. Since we have your father’s results, we know you are at 50% risk to inherit the same pathogenic variant that he was found to have in the APC gene. Genetic testing will look at your APC gene to see if that same pathogenic variant is present. If it is, this would confirm a diagnosis of FAP in you, and you would be recommended to start screening. If it is not found, you would not be at risk for this condition and would not need a colonoscopy until the typical age of 45.”  Pt: “Oh, that is a lot easier than I thought. Just a spit test and I would know?”  *Feeling Reflection*  GC: “Yes, then you can stop worrying and know once and for all. Probably a good idea”  Pt: I guess we can get this over with. “  *Content Reflection*  GC: “If you’d like, we can collect a sample today and send it to the lab for testing. Otherwise, when you’re ready to move forward with testing, I will mail you a saliva kit that you mail back. Either way, we’ll meet to go over your results, and discuss potential screening and follow-up.”  Pt: “So I don’t have to decide today? I guess I should talk to my dad again. Let me think about it Will you just mail it to me instead? I think I need more time.  GC: Ok, but let’s get your consent now and review the legal documents before you go.  Pt: Okay, I guess.  **Outcome:** GC reviews informed consent and discusses GINA. Patient provides written consent. GC mails the patient a saliva kit, and after 3 weeks of not receiving it back from the patient, the GC follows up with him but never hears back. |  | GC: “Let me review what I see in your record. Your father had colon cancer at age 33 and tested positive for FAP. It looks like because of that you’ve had a thyroid ultrasound scan a couple years ago that was normal, but you’ve not had a colonoscopy yet. And you haven’t had a genetic test.”  Pt: “Right. I’m just not sure I need that colon test and I don’t want to deal with the genetic test. I was hoping to wait until after college to handle these things. The test scares me”  *Rolling with Resistance*  GC: “Your plan was to get through college before undergoing more medical procedures. You don’t feel like dealing with these things now. It’s something that is in the back of your head, but you aren’t quite ready to deal with it all.”  Pt: “Yes, I’m just trying to focus on school. I know I probably should get these things done, but ugh. I remember my dad telling us stories about the prep. He said it is nasty.  *Content and Feeling Reflection*  GC: “So you are really put off by what you have heard about the colonoscopy prep.  Pt: Yeah, you have to drink a gallon of salty horrible water  GC: And that sounds miserable.  Pt: Yeah  GC: You mentioned being scared about the genetic test. Tell me more about that.”  Pt: “Well I think I should just wait. What’s the point of doing it now? Would it have helped my father? Plus, I don’t know if I can handle a lifetime of colonoscopies if they find something.  *Feeling Reflection*  GC: “So you are not sure how the genetic test would benefit you and if they find something it would mean having an annoying colonoscopy every year. Just seems easier to push it off for a while”  Pt: “Yeah, I am not sure how I would handle a positive result. Seems like it would change everything. My dad tried to stay positive about it. He’s always said, ‘Yeah, I have a bag <points down, to a bag>, but I also have a life!’ I admire that he can be so positive, but I am not sure I can be as positive as him”  *Content Reflection*  GC: “So part of it is that you are not sure you can be as strong as your dad and not let it cloud everything in your life.”  Pt: Yeah, I just don’t know how I will handle a positive result.  *Change Talk Reflection*  GC: “Perhaps knowing your likelihood of having FAP might help you decide if you want to go ahead with all of this.”  Pt: “Yeah, I guess.  *Elicit permission*  GC: “If it’s OK with you, I can share what I know.”  Pt: “Sure.”  *Provide new information. Elicit feedback.*  GC: “Since we have your father’s results, we know you have a 50% chance of having the same genetic mutation or pathogenic variant that causes FAP. The gene they look at is called APC. If you have the variant this would mean you have FAP. If it is not found, you would not be at risk for this condition. We usually get results back in 3-4 weeks. If you are negative, then you wouldn’t have to get a colonoscopy until you are 45 or so. What do you make of this?”  Pt: 50/50…that’s a bit better than I thought. I thought it was like 90%”  *Feeling Reflection*  GC: So, you are a bit relieved about the odds.  Pt: “I guess. Still sucks but I guess it isn’t inevitable. And, I know at some point I have to find out. It would be nice to stop obsessing over it”  *Change talk Reflection*  GC: “You’re starting to see how knowing your status can help you.”  Pt: “Yeah, I guess worrying isn’t helping me. And it would be great if I could avoid having colonoscopies every year.”  *Action Reflection*  GC: “Given what you said, it sounds like you might be ready to hear more about APC gene testing. It is still of course completely up to you if you want to have the test.”  Pt: “Yes, I think knowing my status will help me be less anxious about this. I think about it a lot. I guess if I do have the FAP, at least I have my dad around to walk me through it. If I wait too long, he could be gone.”  *Meaning Reflection*  GC: “Having your dad as a support will be valuable regardless of your results. That’s a reason to get it done sooner.”  Pt: “Definitely. And I know he’ll feel proud of me for getting tested. He might be less worried if I do. Can I do it today?”  *Providing new information*  GC: “If you’d like, we can collect a sample today and send it to the lab for testing. Otherwise, when you’re ready to move forward with testing, I will mail you a saliva kit that you mail back. Either way, we’ll meet to go over your results, and discuss potential screening and follow-up.”  Pt: “I think I can do that.”  *Eliciting permission to summarize*  GC: Now, I’d like to summarize our visit today, if that’s OK.”  Pt: “Yeah, we did discuss a lot.”  *Summarize*  GC: “You’ve put off getting a GT for a while in part because you were scared to find out if you had FAP like your dad, which would disrupt your life… lead to a lot of extra tests… on the other hand, you are starting to think that it would be good to know, and if something if found, you would have your dad around to help you through it…”  Pt: “Yeah, that’s about right…I think maybe it's time.”  *Open question*  GC: “What, if any, other questions or concerns might you have before I go over the consent form and documentation?”  Pt: Nothing else. I think I should do it.  **Outcome:** GC reviews informed consent and discussed GINA. Patient signed consent and completed the saliva kit before ending the visit. He completed his follow-up visit with the GC to discuss his test results. |

In the encounter on the left, the counselor may have prematurely pushed the patient to decide before his ambivalence was resolved and his motivation crystallized. He wasn’t quite ready, although he tentatively agreed to get tested. We refer to this as a “pseudo yes”. In the traditional GC case, the counselor focuses more on providing education related to the benefits and importance of genetic testing for FAP. In the MI version, the GC spent extra time and effort building a stronger “WHY”, exploring why the test may be important to him and allowing him to make these connections himself. Here the patient was more fully engaged in the decision and more committed to taking action. He followed through with the test and post-test consultation. While the MI encounter ran a little longer, the added rapport and motivation building produced a better outcome.
